# Contemporary Management of Acute Coronary Syndromes in the Cardiac Intensive Care Unit: From Rapid Diagnosis to Early Secondary Prevention

**DOI:** 10.3390/jcdd13080393

**Published:** 2026-08-14

**Authors:** Domenico Mario Giamundo, Giuseppe Andò, Mamas A. Mamas, Atul Pathak, Salvatore De Rosa, Marco Bernardi, Stefano Figliozzi, Mirvat Alasnag, Dan Atar, Elad Asher, Pierre Sabouret

**Affiliations:** 1 Division of Cardiology, Policlinico Casilino, 00169 Rome, Italy; 2 Department of Clinical and Experimental Medicine, University of Messina, 98125 Messina, Italy; giuseppe.ando@unime.it; 3 Keele Cardiovascular Research Group, Department of Cardiology, Keele University, Newcastle ST5 5BG, UK; m.mamas@keele.ac.uk; 4 National Institute of Cardiology, Cardiovascular Surgery and Interventional Cardiology (INCCI), L-1210 Luxembourg City, Luxembourg; pathak.atul@incci.lu; 5 Department of Medical and Surgical Sciences, Magna Graecia University, 88100 Catanzaro, Italy; saderosa@unicz.it; 6 Department of Clinical, Internal Medicine, Anesthesiology and Cardiovascular Sciences, Sapienza University of Rome, 00161 Rome, Italy; m.bernardi@ausl.latina.it; 7 Department of Biomedical Sciences, Humanitas University, Via Rita Levi Montalcini 4, Pieve Emanuele, 20090 Milano, Italy; stefano.figliozzi@humanitas.it; 8 Cardiac Center, King Fahd Armed Forces Hospital, Jeddah 9862, Saudi Arabia; mirvat@jeddacath.com; 9 Division of Cardiology, Oslo University Hospital Ulleval, 0450 Oslo, Norway; dan.atar@medisin.uio.no; 10 Jesselson Heart Center, Shaare Zedek Medical Center, The Eisenberg R&D Authority and Faculty of Medicine, Hebrew University of Jerusalem, Jerusalem 9103102, Israel; easher@szmc.org.il; 11 ACTION Study Group, Inserm UMRS1166, Heart Institute, Pitié-Salpetriere Hospital, Sorbonne University, 47-83 Bd de L’Hôpital, 75013 Paris, France; pierre.sabouret@action-coeur.org

**Keywords:** acute coronary syndrome, cardiac intensive care unit, high-sensitivity cardiac troponin, antithrombotic therapy, coronary revascularisation, cardiogenic shock, secondary prevention

## Abstract

Management of acute coronary syndromes (ACS) in the cardiac intensive care unit (CICU) requires rapid diagnosis, timely reperfusion or invasive assessment, appropriate antithrombotic therapy, and early recognition of haemodynamic, electrical, and mechanical complications. This narrative review examines contemporary evidence across ST-segment elevation and non-ST-segment elevation presentations, with emphasis on decisions during hospitalisation and early secondary prevention. High-sensitivity cardiac troponin algorithms support accelerated assessment of suspected non-ST-segment elevation ACS, whereas next-generation assays require further implementation validation. Twelve-month dual antiplatelet therapy remains the default after ACS in patients without high bleeding risk; abbreviated regimens, de-escalation, cangrelor, and combined antiplatelet–anticoagulant treatment are selective strategies. Contemporary care also includes risk-based invasive timing, complete revascularisation in suitable haemodynamically stable patients, culprit-lesion-only initial PCI in cardiogenic shock, and individualised management of frailty and renal impairment. High-intensity statin therapy, with early ezetimibe when needed, is guideline-supported. Early PCSK9 inhibition and low-dose colchicine are selective strategies, whereas SGLT2 inhibitors and GLP-1RAs are established for specific comorbid indications. hs-cTnT Gen 6 and AI-assisted tools remain evidence-evolving, while multiomics and targeted anti-inflammatory therapies remain investigational. Clear separation of guideline-supported, selective, evidence-evolving, and investigational approaches is essential for clinically appropriate ACS care.

## 1. Introduction

Acute coronary syndromes (ACS) encompass a heterogeneous spectrum ranging from uncomplicated myocardial infarction to cardiogenic shock, malignant ventricular arrhythmias, and mechanical complications. Contemporary management in the cardiac intensive care unit (CICU) therefore requires the rapid integration of diagnostic assessment, coronary reperfusion or invasive treatment, antithrombotic therapy, haemodynamic support, management of comorbidities, and initiation of secondary prevention [1,2,3].

High-sensitivity cardiac troponin assays and accelerated diagnostic algorithms have transformed the assessment of suspected non-ST-segment elevation ACS, while advances in reperfusion and revascularisation have improved the treatment of ST-segment elevation myocardial infarction and high-risk non-ST-segment elevation presentations [1,4,5]. At the same time, increasingly complex CICU populations require greater attention to multivessel coronary disease, shock, electrical and mechanical complications, renal dysfunction, frailty, bleeding risk, and concomitant oral anticoagulation [1,2,6].

Secondary prevention should begin during the index hospitalisation. Intensive lipid-lowering and guideline-directed antithrombotic treatment are guideline-supported components of care. Early PCSK9 inhibition and low-dose colchicine are selective strategies; SGLT2 inhibitors and GLP-1RAs are established for specific comorbid indications; next-generation biomarkers and AI-assisted tools remain evidence-evolving; and multiomics and targeted anti-inflammatory therapies remain investigational. Evidence-evolving and investigational approaches should not be presented as equivalent to interventions supported for routine clinical practice.

**Figure 1:**
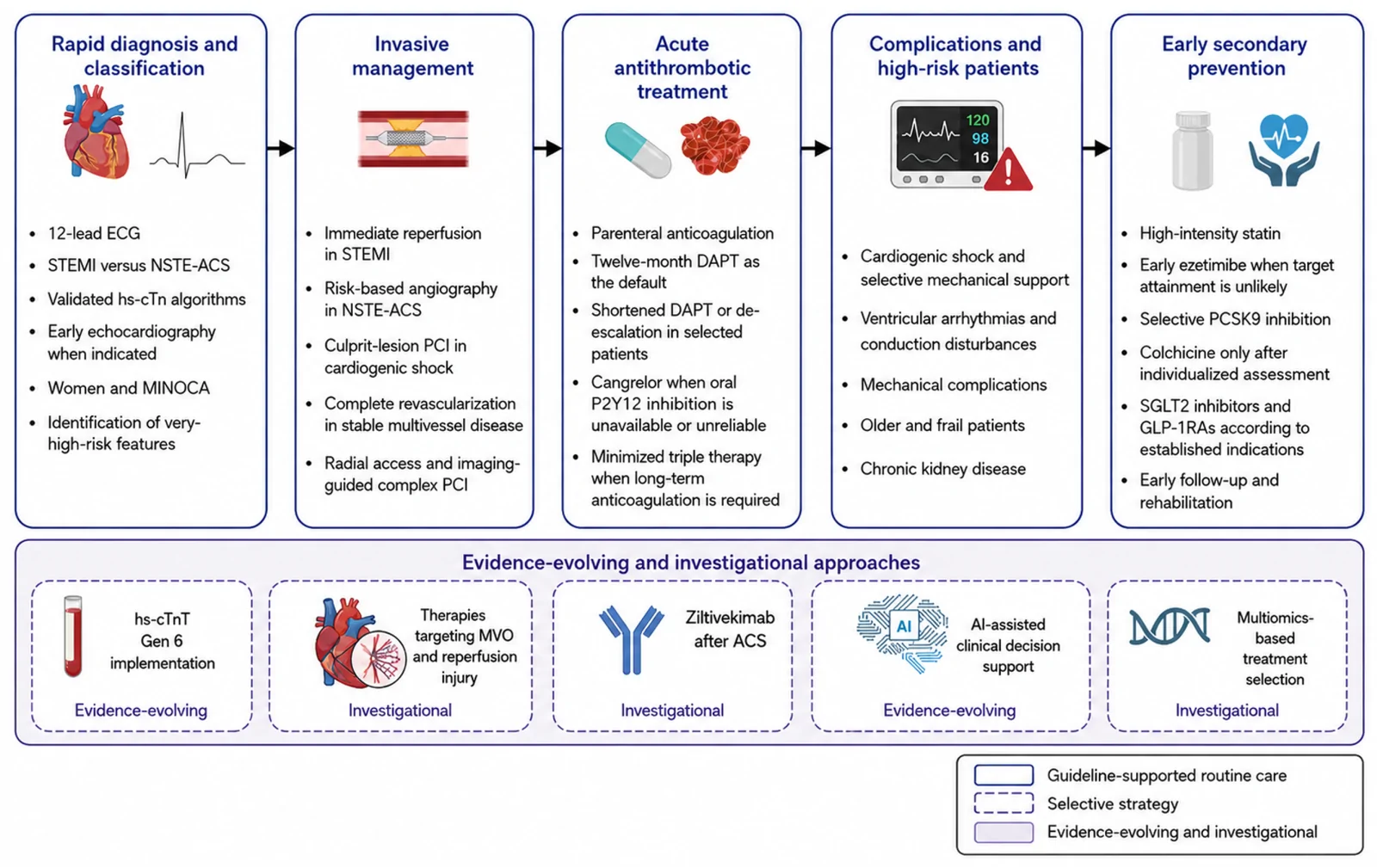
Evidence-based management of acute coronary syndromes in the cardiac intensive care unit. The figure summarises the main diagnostic and therapeutic decisions relevant to patients with acute coronary syndromes (ACS) during hospitalisation and the early post-acute phase. The upper pathway includes rapid diagnosis and classification, invasive management, acute antithrombotic treatment, management of complications and high-risk patients, and secondary prevention initiated during hospitalisation. Guideline-supported interventions are distinguished from selective strategies requiring individualised assessment, while the lower section identifies evidence-evolving and investigational approaches. Abbreviations: ACS, acute coronary syndrome; AI, artificial intelligence; CKD, chronic kidney disease; DAPT, dual antiplatelet therapy; ECG, electrocardiogram; GLP-1RA, glucagon-like peptide-1 receptor agonist; hs-cTn, high-sensitivity cardiac troponin; hs-cTnT, high-sensitivity cardiac troponin T; MINOCA, myocardial infarction with non-obstructive coronary arteries; MVO, microvascular obstruction; NSTE-ACS, non-ST-segment elevation acute coronary syndrome; PCI, percutaneous coronary intervention; PCSK9, proprotein convertase subtilisin/kexin type 9; SGLT2, sodium–glucose cotransporter 2; STEMI, ST-segment elevation myocardial infarction.

This narrative review examines contemporary evidence across the full spectrum of ACS, focusing on decisions relevant to the CICU and early post-acute phase. Particular attention is given to distinguishing guideline-supported care from selective strategies and from interventions that remain evidence-evolving or investigational. The principal diagnostic and therapeutic pathways are summarised in Figure 1.

## 2. Methods

This narrative review was based on a targeted search of PubMed/MEDLINE and relevant international guideline documents, updated to 31 July 2026. The search focused primarily on publications from the preceding seven years, while earlier landmark studies were retained when essential to the development of current practice. Search terms covered ACS diagnosis, antithrombotic treatment, coronary revascularisation, cardiogenic shock, mechanical circulatory support, complications, special populations, and early secondary prevention.

Priority was given to European and North American clinical practice guidelines, randomised controlled trials, large prospective studies, systematic reviews, and meta-analyses relevant to ACS management during hospitalisation and the early post-acute phase. Evidence from broader cardiovascular populations was included only when it had a direct implication for ACS care or concerned an established comorbid indication.

ClinicalTrials.gov and official trial-sponsor communications were also reviewed for ongoing studies and recently announced results not yet available as peer-reviewed publications; these sources are explicitly identified as such in the text. Guideline-supported interventions were distinguished from selective, evidence-evolving, or investigational approaches. No formal systematic review, risk-of-bias assessment, or quantitative evidence synthesis was performed.

## 3. Rapid Diagnosis and Early Risk Stratification

### 3.1. High-Sensitivity Cardiac Troponin in Suspected ACS

The role of cardiac troponin differs across the ACS spectrum. In patients with persistent ST-segment elevation or other electrocardiographic evidence of acute coronary occlusion, reperfusion should not be delayed while awaiting biomarker results. High-sensitivity cardiac troponin (hs-cTn) measurement is instead central to the assessment of suspected non-ST-segment elevation ACS (NSTE-ACS) [1].

According to the Fourth Universal Definition of Myocardial Infarction, myocardial injury is defined by at least one cardiac troponin concentration above the 99th-percentile upper reference limit and is considered acute when a rise and/or fall is documented. Myocardial infarction additionally requires evidence of acute myocardial ischaemia derived from symptoms, electrocardiography, imaging, or the identification of coronary thrombosis [4]. This distinction is particularly important in the CICU, where troponin elevation may accompany heart failure, pulmonary embolism, sepsis, tachyarrhythmias, myocarditis, renal dysfunction, and other critical illnesses without acute coronary atherothrombosis.

Current European recommendations support validated assay-specific 0/1 h or 0/2 h algorithms in patients with suspected NSTE-ACS [1]. These pathways combine the concentration at presentation with its early absolute change to classify patients into rule-out, observe, or rule-in categories. When applied to appropriate populations, they permit earlier diagnostic classification than conventional 3–6 h sampling and may reduce unnecessary observation and emergency department length of stay [1,5,7].

Biomarker findings must nevertheless be integrated with the clinical presentation. An elevated hs-cTn concentration or rule-in classification does not identify the mechanism of myocardial injury, while a rule-out result does not exclude unstable angina or other clinically relevant causes of chest pain. Further evaluation may therefore be required in patients with recurrent symptoms, ischaemic electrocardiographic changes, known coronary disease, chronic troponin elevation, renal dysfunction, or results within the observe zone. Serial electrocardiography, echocardiography, coronary computed tomography angiography, stress imaging, or invasive angiography should be selected according to clinical risk and the suspected mechanism [1,4,5].

### 3.2. Next-Generation hs-cTnT Assays: Diagnostic Potential and Current Limitations

The sixth-generation high-sensitivity cardiac troponin T assay (hs-cTnT Gen 6) was developed to improve analytical performance at low troponin concentrations and reduce susceptibility to assay interference. Initial diagnostic studies have evaluated assay-specific thresholds and accelerated rule-in and rule-out pathways, reporting high diagnostic accuracy in patients with suspected myocardial infarction [8].

These findings establish analytical and diagnostic potential but do not demonstrate improved clinical outcomes or healthcare delivery. PERFORM-TSIX was designed as a large prospective international cohort to evaluate the assay at different sampling times and validate thresholds for accelerated algorithms [9]. However, the cited publication describes the study design rather than completed clinical results.

It therefore cannot support claims that hs-cTnT Gen 6 reduces repeat sampling, shortens hospital stay, accelerates treatment, or improves prognosis. These outcomes require prospective implementation studies directly comparing the assay with established hs-cTn pathways. At present, hs-cTnT Gen 6 should be classified as evidence-evolving rather than as a guideline-supported strategy for improving CICU workflow or patient outcomes.

### 3.3. Women Presenting with Acute Coronary Syndromes

Women with suspected ACS should receive the same timely, risk-based diagnostic assessment as men. Chest pain or discomfort remains the most frequent presenting symptom, although dyspnoea, nausea, fatigue, epigastric discomfort, and pain involving the back, shoulder, neck, or jaw may coexist and contribute to delayed recognition. Decisions regarding invasive or non-invasive assessment should therefore be based on symptoms, electrocardiographic findings, haemodynamic status, and ischaemic risk rather than sex alone [1,10].

High-sensitivity cardiac troponin concentrations are generally lower in women. Assay-specific sex-specific 99th-percentile upper reference limits increase the identification of myocardial injury and myocardial infarction, although improved diagnostic classification has not consistently translated into better outcomes when subsequent investigation and treatment remain unequal [11]. Troponin results should therefore be interpreted together with serial changes and the overall clinical presentation.

Non-atherosclerotic mechanisms, including spontaneous coronary artery dissection, coronary spasm, embolism, and myocardial infarction with non-obstructive coronary arteries, are more frequent in younger and premenopausal women. Recognising the underlying mechanism is essential because treatment developed for atherosclerotic plaque rupture may not be appropriate in every case [12].

### 3.4. Myocardial Infarction with Non-Obstructive Coronary Arteries

Myocardial infarction with non-obstructive coronary arteries (MINOCA) is a working diagnosis rather than a final disease entity. It requires fulfilment of the universal criteria for acute myocardial infarction, the absence of stenosis ≥50% in any major epicardial coronary artery, and no immediately apparent alternative explanation for the presentation [1,13]. Potential ischaemic mechanisms include angiographically unrecognised plaque disruption, epicardial or microvascular spasm, spontaneous coronary artery dissection, coronary thromboembolism, and microvascular dysfunction. Myocarditis, Takotsubo syndrome, pulmonary embolism, sepsis, and other causes of myocardial injury should instead be excluded.

Identification of non-obstructive coronary arteries should be followed by reassessment of the angiogram and ventricular function and, whenever feasible, early cardiac magnetic resonance. Optical coherence tomography or intravascular ultrasound may reveal plaque disruption, intramural haematoma, or subtle coronary dissection, while coronary functional testing may be considered in selected stable patients with suspected epicardial or microvascular vasomotor abnormalities [1,13,14]. Combining intracoronary imaging with cardiac magnetic resonance can identify the underlying mechanism in a substantial proportion of patients.

Treatment should be directed at the identified cause rather than uniformly extrapolated from obstructive atherosclerotic myocardial infarction. Antiplatelet and lipid-lowering therapies are appropriate when plaque disruption is demonstrated or strongly suspected, whereas coronary spasm, embolism, and spontaneous coronary artery dissection require mechanism-specific management.

In the PROMISE trial, a comprehensive diagnostic work-up followed by aetiology-guided treatment improved angina-related health status at 12 months compared with standard care [15]. However, only 92 patients with confirmed MINOCA were included, and the study was not powered to assess major cardiovascular events. Its findings should therefore be considered preliminary and require confirmation in larger trials.

## 4. Acute Antithrombotic Management

Antithrombotic treatment should balance protection from recurrent ischaemic events against bleeding risk, taking into account the ACS presentation, invasive strategy, coronary anatomy, renal function, and any indication for long-term oral anticoagulation [1,12]. Individualisation should adapt, rather than replace, guideline-directed treatment.

### 4.1. Default DAPT and Individualised Treatment Duration

Dual antiplatelet therapy (DAPT) with aspirin and an oral P2Y12 receptor inhibitor for 12 months remains the default after ACS in patients without high bleeding risk [1,12]. Ticagrelor or prasugrel is generally preferred after PCI, while clopidogrel remains appropriate when potent P2Y12 inhibition is contraindicated, poorly tolerated, unavailable, or unsuitable because of bleeding risk or concomitant anticoagulation.

Shortened DAPT, aspirin withdrawal, and P2Y12 inhibitor de-escalation are selective strategies for appropriate patients rather than routine approaches. In TWILIGHT, aspirin withdrawal after 3 event-free months reduced clinically relevant bleeding in high-risk patients undergoing PCI without a significant increase in the composite ischaemic endpoint [16]. The trial population was selected after successful completion of the initial DAPT period, limiting generalisation to all patients with ACS.

MASTER DAPT showed that, in high-bleeding-risk patients who had completed 1 month of DAPT, abbreviated treatment reduced bleeding and was non-inferior for net and major clinical events [17]. Guided de-escalation from prasugrel to clopidogrel in TROPICAL-ACS provided another option for reducing treatment intensity in selected patients [18]. DAPT duration should therefore be reassessed according to evolving ischaemic and bleeding risks, particularly after bleeding, recurrent ischaemia, renal deterioration, newly diagnosed atrial fibrillation, or the need for surgery [1,12].

### 4.2. Cangrelor in Selected Patients Undergoing PCI

Oral P2Y12 inhibition may be delayed or unreliable in patients with vomiting, opioid administration, cardiogenic shock, cardiac arrest, mechanical ventilation, or inability to swallow. Cangrelor, a direct and reversible intravenous P2Y12 inhibitor, may be considered in P2Y12 inhibitor-naïve patients undergoing PCI when oral treatment has not been administered, cannot be given, or is unlikely to be adequately absorbed [1,19].

In CHAMPION PHOENIX, cangrelor reduced the 48 h composite of death, myocardial infarction, ischaemia-driven revascularisation, or stent thrombosis compared with clopidogrel, without a significant increase in severe bleeding [19]. However, it was not primarily compared with contemporary prasugrel- or ticagrelor-based treatment, and routine use in all patients undergoing PCI is not supported. Cangrelor should therefore remain a selective strategy rather than routine therapy.

Its rapid offset also permits bridging in selected patients requiring interruption of oral P2Y12 inhibition before cardiac surgery. BRIDGE demonstrated maintained platelet inhibition without a significant excess of major CABG-related bleeding, but was primarily a pharmacodynamic rather than an ACS outcome trial [20]. Bridging should therefore remain a specialised strategy.

### 4.3. Anticoagulation and Long-Term Oral Anticoagulation

Parenteral anticoagulation is a guideline-supported component of acute ACS treatment and should be selected according to the presentation, invasive strategy, renal function, and bleeding risk [1]. Unfractionated heparin remains the standard procedural anticoagulant during primary or urgent PCI, while enoxaparin or bivalirudin may be considered in appropriate settings. When fondaparinux has been given before PCI, additional unfractionated heparin is required during the procedure [1]. Routine continuation after uncomplicated PCI is unnecessary unless another indication is present.

Patients requiring long-term oral anticoagulation, most commonly for atrial fibrillation, are at particularly high bleeding risk. Prolonged triple therapy with aspirin, a P2Y12 inhibitor, and an oral anticoagulant should be avoided whenever possible. The default European strategy is triple therapy for up to 1 week, followed by an oral anticoagulant plus clopidogrel for up to 12 months; triple therapy may be extended to 1 month when the risk of stent thrombosis clearly outweighs bleeding risk [1].

The 2025 North American guideline similarly favours early aspirin withdrawal, generally within 1–4 weeks after PCI, followed by an oral anticoagulant and a P2Y12 inhibitor [12]. Both approaches therefore minimise triple-therapy duration. A direct oral anticoagulant is generally preferred to a vitamin K antagonist when eligible, with dose and indication reassessed according to renal function and bleeding risk before discharge.

## 5. Invasive Management and Revascularisation

Revascularisation remains central to the management of ACS, but its timing and extent should be determined by the clinical presentation, haemodynamic status, ischaemic risk, coronary anatomy, and comorbidities. The contemporary invasive strategy should therefore distinguish patients requiring immediate reperfusion from those in whom coronary angiography can be performed during hospitalisation according to risk. The same distinction is essential when deciding whether treatment should be limited to the culprit lesion or extended to achieve complete revascularisation [1,12].

### 5.1. Reperfusion in STEMI and Timing of the Invasive Strategy in NSTE-ACS

In patients with a working diagnosis of ST-segment elevation myocardial infarction (STEMI), reperfusion should not be delayed while awaiting cardiac troponin results. Primary percutaneous coronary intervention (PCI) is preferred when it can be performed within 120 min of the electrocardiographic diagnosis. When timely primary PCI is unavailable, fibrinolysis should be administered promptly to eligible patients presenting within 12 h of symptom onset, followed by transfer to a PCI-capable centre [1].

Persistent symptoms, haemodynamic or electrical instability, or inadequate ST-segment resolution after fibrinolysis require immediate rescue PCI. After apparently successful fibrinolysis, routine coronary angiography should be performed within 2–24 h. In late presenters, invasive treatment remains indicated when ongoing ischaemia, haemodynamic instability, life-threatening arrhythmia, or a mechanical complication is present. Conversely, routine PCI of a persistently occluded infarct-related artery is not recommended in stable asymptomatic patients presenting more than 48 h after symptom onset [1].

In non-ST-segment elevation ACS (NSTE-ACS), the timing of angiography should be determined by clinical risk rather than troponin elevation alone. An immediate invasive strategy is required in patients with haemodynamic instability or cardiogenic shock, recurrent or refractory chest pain, acute heart failure due to ongoing ischaemia, life-threatening arrhythmias, mechanical complications, or recurrent dynamic electrocardiographic changes suggesting acute coronary occlusion [1].

Patients with confirmed NSTEMI, a GRACE score above 140, dynamic ST-segment or T-wave changes, or transient ST-segment elevation should undergo an invasive strategy during hospitalisation and may be considered for angiography within 24 h [1]. However, randomised trials have failed to demonstrate a consistent reduction in death or myocardial infarction with an early rather than delayed strategy in unselected NSTE-ACS populations, although earlier intervention may reduce recurrent ischaemia and shorten hospitalisation. The benefit of early angiography is therefore most plausible in patients with high-risk features.

The 2025 North American guideline similarly recommends an invasive approach during hospitalisation for patients at intermediate or high ischaemic risk, while either a routine invasive strategy or further non-invasive risk stratification may be used in lower-risk patients [12]. Both European and North American guidance therefore support risk-based rather than indiscriminate early angiography.

### 5.2. Multivessel Coronary Disease

Multivessel coronary disease is frequent in ACS and is associated with an increased risk of recurrent events. In haemodynamically stable patients with STEMI who have undergone successful culprit-lesion PCI, complete revascularisation of suitable significant non-culprit lesions is recommended. COMPLETE showed that staged non-culprit PCI reduced cardiovascular death or myocardial infarction and ischaemia-driven revascularisation compared with culprit-lesion-only treatment [21].

BIOVASC, which enrolled patients across the ACS spectrum, demonstrated that immediate complete revascularisation was non-inferior to a staged strategy and was associated with fewer early myocardial infarctions and unplanned ischaemia-driven procedures. MULTISTARS AMI similarly established the non-inferiority of immediate multivessel PCI in haemodynamically stable patients with STEMI [22,23]. These findings support both immediate and staged approaches but do not establish a single strategy for every patient.

The choice should account for clinical stability, lesion complexity, renal function, contrast and radiation exposure, procedural duration, operator experience, and the possible need for surgical revascularisation. A staged procedure may be preferable after a prolonged culprit intervention, in patients with renal impairment or complex non-culprit disease, or when the significance of intermediate lesions remains uncertain.

The 2023 European guideline permits complete revascularisation during the index procedure, the same hospitalisation, or within 45 days, without a firm preference for immediate or staged PCI. The 2025 North American guideline also permits both strategies but favours single-session multivessel PCI when clinically and anatomically appropriate [1,12].

Evidence is less definitive in NSTE-ACS because no dedicated randomised trial has compared complete with culprit-only PCI exclusively in this population. Complete revascularisation should nevertheless be considered, with the choice between multivessel PCI and coronary artery bypass grafting based on coronary complexity, left main involvement, diabetes, ventricular function, comorbidities, surgical risk, and patient preferences [1,12]. Heart Team discussion is appropriate when the optimal method is uncertain.

These recommendations do not apply unchanged to patients with cardiogenic shock, in whom the initial procedure should generally be restricted to the infarct-related artery, as discussed in Section 6.1.

### 5.3. Procedural Considerations

Procedural strategy may also influence outcomes in patients undergoing invasive treatment for ACS. Radial arterial access is preferred over femoral access whenever feasible because it reduces access-site bleeding and vascular complications. Intravascular imaging should be used to optimise PCI in complex coronary lesions, including left main disease, bifurcations, long lesions, uncertain stent expansion, or suspected procedural complications [1,12].

These techniques should be regarded as components of high-quality PCI rather than as isolated technological advances. Their use should be adapted to the patient’s anatomy, haemodynamic condition, bleeding risk, and the urgency of the procedure.

### 5.4. Microvascular Obstruction and Ischaemia–Reperfusion Injury

Restoration of epicardial coronary flow does not invariably result in adequate myocardial reperfusion. Microvascular obstruction (MVO) after primary PCI may arise from distal embolisation, endothelial and interstitial oedema, vasoconstriction, intramyocardial haemorrhage, inflammation, and microvascular thrombosis. When detected by cardiac magnetic resonance, MVO is associated with larger infarct size, adverse left ventricular remodelling, heart failure, and an unfavourable prognosis after STEMI [24]. Despite its clinical relevance, no adjunctive pharmacological or device-based treatment has demonstrated sufficiently consistent benefit to support routine use.

The immediate management of angiographic slow flow or no-reflow should first exclude mechanical causes, including dissection, residual epicardial obstruction, coronary spasm, or distal embolisation. Haemodynamic optimisation and selective intracoronary vasodilator therapy may be considered, while glycoprotein IIb/IIIa inhibition may be used as a bailout strategy in the presence of a large thrombus burden or an acute thrombotic complication [1]. However, evidence that these measures reduce infarct size or improve long-term outcomes remains limited.

Several strategies intended to prevent or reduce MVO have produced neutral or inconsistent results. Intracoronary alteplase did not reduce MVO in T-TIME and did not improve myocardial reperfusion or short-term clinical outcomes in STRIVE, even among patients with a high thrombus burden [25,26]. Similarly, cyclosporine in CIRCUS and colchicine in COVERT-MI did not reduce adverse remodelling or infarct size, while small studies of interleukin-1 blockade have provided only exploratory signals [27,28,29]. Mechanical left ventricular unloading before reperfusion did not improve infarct size or clinical outcomes in STEMI-DTU, and other approaches, including supersaturated oxygen delivery, remain supported mainly by selected populations or surrogate endpoints [30,31].

Prompt epicardial reperfusion, avoidance of treatment delays, optimal PCI technique, and early recognition of no-reflow therefore remain the foundations of care. Future trials should identify patients at greatest risk of MVO and determine whether improvement in imaging or physiological endpoints translates into fewer episodes of heart failure, recurrent cardiovascular events, or death. Until such evidence becomes available, therapies directed specifically at MVO and ischaemia–reperfusion injury should remain classified as investigational.

## 6. Complicated ACS in the Cardiac Intensive Care Unit

Acute coronary syndromes complicated by cardiogenic shock, malignant arrhythmias, or acute mechanical complications require immediate recognition and coordinated management in the CICU. These conditions should be addressed separately from uncomplicated ACS because diagnostic and therapeutic priorities are dominated by haemodynamic stabilisation, emergency revascularisation, and correction of the underlying complication.

### 6.1. Cardiogenic Shock and Temporary Mechanical Circulatory Support

Cardiogenic shock is a heterogeneous syndrome in which primary cardiac dysfunction causes systemic hypoperfusion and progressive organ injury. Hypotension is common but is not required, as clinically relevant hypoperfusion may precede a marked fall in arterial pressure. Assessment should include mental status, peripheral perfusion, urine output, lactate, acid–base balance, renal and hepatic function, and the requirement for vasoactive treatment [32]. Serial evaluation is essential because the severity and haemodynamic phenotype may evolve rapidly.

Urgent echocardiography should assess left and right ventricular function, filling conditions, and possible mechanical complications. Invasive haemodynamic monitoring may be considered when the shock phenotype remains uncertain, right ventricular or mixed failure is suspected, or the response to initial treatment is inadequate [32]. Supportive care should restore oxygenation and organ perfusion while avoiding indiscriminate fluid administration and excessive doses of vasopressors or inotropes, none of which has demonstrated a consistent survival advantage.

Emergency coronary revascularisation remains the only intervention with an established survival benefit in myocardial infarction-related cardiogenic shock. In patients with multivessel disease, the initial procedure should generally be limited to the infarct-related artery. CULPRIT-SHOCK showed that culprit-lesion-only PCI, with the option of staged revascularisation, reduced the 30-day composite of death or renal replacement therapy compared with immediate multivessel PCI [33]. Non-culprit disease may be addressed after haemodynamic stabilisation, while emergency coronary artery bypass grafting remains an option when PCI is not feasible.

Temporary mechanical circulatory support may be considered when hypoperfusion persists despite revascularisation and appropriate medical treatment. Device selection should reflect the predominant ventricular failure, shock severity and trajectory, oxygenation, vascular anatomy, neurological prognosis, and the likelihood of recovery or transition to a definitive therapy. Whenever feasible, these decisions should be made by a multidisciplinary shock team and should not delay correction of a reversible cause [32].

Routine intra-aortic balloon pump use is not recommended in infarct-related cardiogenic shock without a mechanical complication, as IABP-SHOCK II demonstrated no mortality benefit [34]. Balloon counterpulsation may nevertheless be considered as a bridge in selected patients with acute mitral regurgitation, ventricular septal rupture, or another mechanical complication. Similarly, ECLS-SHOCK showed that routine early venoarterial extracorporeal support did not reduce 30-day mortality and increased bleeding and vascular complications [35]. VA-ECMO should therefore be reserved for carefully selected patients with refractory shock, severe biventricular or respiratory failure, and a potentially reversible condition or realistic bridge strategy.

DanGer-Shock provided evidence that early microaxial flow-pump support can reduce 180-day mortality in selected patients with STEMI-related cardiogenic shock, but at the cost of more major bleeding, limb ischaemia, and renal complications [36]. These findings support selective rather than routine use. Overall, the decision to initiate, escalate, wean, or discontinue mechanical support should balance the probability of haemodynamic recovery against device-related harm and the patient’s overall prognosis.

### 6.2. Electrical Complications

Electrical complications after ACS may reflect ongoing ischaemia, failed reperfusion, extensive myocardial injury, haemodynamic instability, electrolyte abnormalities, or adverse treatment effects. Continuous electrocardiographic monitoring is therefore essential during the early phase after STEMI and in patients with high-risk NSTE-ACS, heart failure, shock, recurrent symptoms, or significant conduction disturbances [1,37].

Ventricular fibrillation and haemodynamically unstable sustained ventricular tachycardia require immediate defibrillation or synchronised cardioversion. Recurrent ventricular arrhythmias should prompt reassessment of coronary patency and consideration of urgent angiography when ongoing ischaemia or recurrent occlusion is suspected [1,37]. Hypoxaemia, acidosis, potassium or magnesium abnormalities, and excessive sympathetic activation should be corrected. Beta-blockers may be used when haemodynamic status permits, while intravenous amiodarone or lidocaine may be considered for recurrent arrhythmias despite electrical treatment [37].

Electrical storm requires a coordinated approach combining repeated cardioversion or defibrillation, correction of reversible triggers, suppression of sympathetic activity, antiarrhythmic treatment, and reassessment of myocardial ischaemia. Deep sedation may reduce adrenergic activation, and catheter ablation may be considered when ventricular tachycardia or ventricular fibrillation remains refractory to revascularisation and medical therapy [37,38]. By contrast, asymptomatic premature ventricular complexes or brief non-sustained ventricular tachycardia during reperfusion generally require no specific treatment; prophylactic antiarrhythmic therapy is not recommended because it has not improved outcomes and may cause proarrhythmia [37].

The timing of sustained ventricular arrhythmias has prognostic implications. Ventricular tachycardia or ventricular fibrillation occurring within the first 48 h is usually related to the acute ischaemic or reperfusion phase and does not, in isolation, establish an indication for an implantable cardioverter–defibrillator. Arrhythmias occurring later than 48 h in the absence of recurrent ischaemia or another reversible cause are associated with a greater risk of recurrence and should prompt assessment for secondary-prevention device therapy [37]. Primary-prevention defibrillator implantation should be deferred during the early post-infarction phase, with reassessment of left ventricular function after recovery and optimisation of medical therapy.

High-grade atrioventricular block accompanying inferior myocardial infarction is often nodal and may resolve after reperfusion, whereas new high-grade block in anterior infarction more commonly reflects extensive His–Purkinje injury [1,39]. Symptomatic bradycardia should initially be managed by correcting ischaemia and other reversible causes. Atropine may be used when hypotension or hypoperfusion is present, and temporary pacing is appropriate when clinically significant bradycardia or high-grade block persists. Permanent pacing should generally be reserved for conduction abnormalities that remain after the acute reversible phase.

New-onset atrial fibrillation may accompany myocardial ischaemia, heart failure, elevated filling pressures, inflammation, or adrenergic activation. Immediate electrical cardioversion is indicated when it contributes to haemodynamic instability. In stable patients, rate or rhythm control should be individualised according to symptoms, ventricular function, arrhythmia duration, and the likelihood of spontaneous resolution. The indication for oral anticoagulation should be assessed separately from the antiplatelet regimen, with careful consideration of the bleeding risk associated with combined therapy [1,40].

### 6.3. Mechanical Complications

Mechanical complications have become less frequent with timely reperfusion but remain associated with rapid haemodynamic deterioration and high mortality. The principal presentations are acute severe mitral regurgitation due to papillary muscle rupture, ventricular septal rupture, and left ventricular free-wall rupture. They should be suspected after myocardial infarction when sudden hypotension, pulmonary oedema, a new murmur, rising lactate, ventricular failure, recurrent chest pain, or pulseless electrical activity develops [1,41].

Urgent transthoracic echocardiography is the first-line diagnostic examination and should assess ventricular function, mitral regurgitation, interventricular shunting, pericardial effusion, and tamponade. Transoesophageal echocardiography may provide more detailed anatomical assessment when transthoracic imaging is inconclusive or intervention is being planned. Diagnostic evaluation and initial stabilisation should proceed in parallel with immediate activation of cardiac surgery and the multidisciplinary shock team [41].

Papillary muscle rupture usually causes abrupt severe mitral regurgitation, pulmonary oedema, and cardiogenic shock. The murmur may be soft or absent when left atrial and ventricular pressures equalise rapidly. Emergency surgery is the definitive treatment, with valve replacement frequently required in complete rupture and repair considered in selected partial ruptures at experienced centres [41]. Afterload reduction, vasoactive treatment, ventilation, and temporary mechanical support may be used as a bridge but should not delay correction.

Ventricular septal rupture produces an acute left-to-right shunt and may progress rapidly to biventricular failure and shock. Surgical repair remains the standard definitive strategy. Immediate intervention is required in patients with refractory heart failure or haemodynamic instability, whereas brief stabilisation may occasionally be considered in carefully selected patients with preserved organ perfusion [41]. Observationally better outcomes after delayed repair should be interpreted cautiously because of substantial survivor-selection bias. Temporary mechanical support may be used as a bridge, while percutaneous closure is reserved mainly for prohibitive surgical risk, selected anatomies, or residual postoperative shunts.

Left ventricular free-wall rupture may present with haemopericardium, tamponade, pulseless electrical activity, or a contained subacute rupture. Emergency surgical repair is required [41]. Pericardial drainage may provide temporary stabilisation in tamponade but should be regarded only as a bridge to surgery, as rapid decompression may increase bleeding through the rupture site. A contained rupture may evolve into a pseudoaneurysm, generally requiring surgical evaluation because of the risk of expansion and rupture.

Across all mechanical complications, pharmacological treatment and temporary circulatory support serve primarily as bridges to definitive correction. Early recognition and rapid transfer to a centre with cardiac surgery, interventional cardiology, advanced imaging, and mechanical-support expertise are essential, and stabilisation attempts should not result in avoidable procedural delay.

## 7. Special Populations

Patients of advanced age and those with frailty, multimorbidity, or chronic kidney disease represent an increasing proportion of the contemporary ACS population. These conditions are associated with higher ischaemic and bleeding risks, greater vulnerability to procedural complications, and frequent uncertainty regarding the net benefit of invasive and pharmacological treatment. Management should therefore preserve access to evidence-based care while incorporating functional status, comorbidities, life expectancy, treatment goals, and patient preferences.

### 7.1. Older and Frail Patients

Chronological age alone should not determine the diagnostic or therapeutic approach to ACS. Older patients differ substantially in functional independence, cognition, comorbidity burden, biological reserve, and life expectancy. Assessment should therefore integrate ischaemic and bleeding risks with frailty, cognitive and functional status, quality of life, procedural risk, and the patient’s preferences [1,42].

High-sensitivity cardiac troponin assays retain diagnostic value in older adults, although chronic myocardial injury and non-coronary causes of troponin elevation are more frequent. Results should therefore be interpreted together with serial changes, symptoms, electrocardiographic findings, imaging, and the presence of acute systemic illness [1]. Similarly, access to primary PCI in STEMI should not be restricted because of age alone, although severe frailty, advanced cognitive impairment, terminal illness, or a low probability of meaningful recovery may influence the proportionality of invasive treatment [1,42].

SENIOR-RITA evaluated 1518 patients aged 75 years or older with NSTEMI who were assigned to a routine invasive or conservative strategy. Over a median follow-up of 4.1 years, routine invasive management did not significantly reduce the primary composite of cardiovascular death or non-fatal myocardial infarction [43]. Non-fatal myocardial infarction occurred less frequently, but cardiovascular mortality was not reduced. These findings do not argue against angiography in older patients with haemodynamic instability, recurrent ischaemia, acute heart failure, malignant arrhythmias, or other very-high-risk features. In clinically stable patients, however, they support an individualised decision rather than routine angiography based solely on age and the diagnosis of NSTEMI.

Frailty is clinically distinct from chronological age. A subsequent SENIOR-RITA analysis found no significant reduction in the primary outcome with the invasive strategy across frailty categories and suggested possible harm among patients with the greatest frailty, although this subgroup finding requires cautious interpretation [44]. A brief validated instrument such as the Clinical Frailty Scale may complement assessment of cognition, comorbidity, independence, procedural risk, and life expectancy. Frailty should inform treatment selection but should not be used as an isolated reason to withhold evidence-based care [42].

Pharmacological treatment should account for body weight, renal function, anaemia, previous bleeding, falls, drug interactions, and the need for oral anticoagulation. Radial access, avoidance of unnecessary triple therapy, and selective use of abbreviated DAPT may reduce bleeding, while undertreatment based solely on age should be avoided. CICU care should also include delirium prevention, early mobilisation, medication reconciliation, and planning of rehabilitation and post-discharge support. In patients with advanced irreversible frailty or comorbidity, a proportionate approach centred on symptom control and quality of life may be appropriate [42].

### 7.2. Chronic Kidney Disease and Renal Impairment

Chronic kidney disease (CKD) is common in patients with ACS and is associated with more extensive coronary disease, recurrent ischaemic events, bleeding, acute kidney injury, and mortality. Despite this higher baseline risk, patients with CKD are less likely to undergo invasive assessment or receive guideline-directed treatment than those with preserved renal function [1,45].

Renal function should be assessed at admission and reassessed during hospitalisation using serum creatinine, estimated glomerular filtration rate, urine output, and the clinical context. A single creatinine value may be misleading in patients with evolving shock, acute heart failure, dehydration, or acute kidney injury. Renally eliminated drugs and exposure to potentially nephrotoxic treatments should therefore be reviewed throughout the hospital course.

Diagnosis may be challenging because baseline troponin concentrations are frequently elevated in CKD. An isolated value above the 99th percentile does not establish acute myocardial infarction; interpretation should incorporate serial changes, clinical evidence of ischaemia, electrocardiography, and imaging. Accelerated hs-cTn algorithms remain applicable, although more patients with renal impairment fall within the observe category and require additional assessment [1].

CKD should not automatically preclude coronary angiography or revascularisation. In STEMI or very-high-risk NSTE-ACS, concern regarding renal deterioration should not delay emergency treatment. In stable patients with advanced CKD, particularly those receiving or approaching kidney replacement therapy, the expected ischaemic benefit should be balanced against bleeding, acute kidney injury, vascular complications, comorbidity burden, and patient preferences [1,46].

Procedural measures should include minimisation of contrast volume and avoidance of unnecessary repeated contrast exposure. Isotonic hydration may be considered in patients with reduced renal function, but should be adapted to ventricular function, filling pressures, and the risk of pulmonary congestion [1]. Excessive routine fluid administration should be avoided.

Antithrombotic agents and doses should be adjusted according to renal function and reassessed when kidney function is unstable. Particular attention is required for parenteral anticoagulants, glycoprotein IIb/IIIa inhibitors, and oral anticoagulants. Unfractionated heparin may offer practical advantages in severe renal impairment because its effect can be monitored and rapidly reversed.

Established secondary-prevention therapies should not be withheld solely because of CKD, although creatinine, potassium, tolerance, and drug interactions require closer monitoring. SGLT2 inhibitors should be used according to established diabetes, heart failure, or CKD indications rather than as routine ACS-specific treatment. Overall, renal impairment should prompt treatment adaptation rather than therapeutic inertia.

## 8. Early Secondary Prevention Initiated During Hospitalisation

### 8.1. Early Intensive Lipid-Lowering Therapy

Patients with ACS are at very high risk of recurrent atherothrombotic events, particularly during the first months after the index event. Lipid-lowering treatment should therefore be initiated or intensified during hospitalisation, and a lipid profile should be obtained early because LDL-cholesterol concentrations may decline during the acute phase [1,47,48].

High-intensity statin therapy remains the foundation of post-ACS lipid management and should be started or continued as early as possible, irrespective of the admission LDL-cholesterol concentration [1,48,49]. PROVE-IT TIMI 22 demonstrated the benefit of intensive rather than moderate statin treatment soon after ACS [49]. European recommendations retain an LDL-cholesterol goal below 55 mg/dL together with a reduction of at least 50% from baseline and encourage early combination therapy when statin monotherapy is unlikely to achieve this target [47,48].

Ezetimibe provides a predictable additional LDL-cholesterol reduction and is generally well tolerated. IMPROVE-IT showed that its addition to statin therapy after ACS produced a modest but significant further reduction in cardiovascular events [50]. Although the trial did not specifically test universal initiation during the first hours of admission, it supports early statin–ezetimibe combination therapy in patients whose baseline LDL-cholesterol, previous treatment, or expected statin response makes target attainment unlikely.

PCSK9 monoclonal antibodies provide profound additional LDL-cholesterol lowering and reduce cardiovascular events in established atherosclerotic disease. FOURIER demonstrated benefit with evolocumab in stable cardiovascular disease, while ODYSSEY OUTCOMES showed that alirocumab reduced recurrent ischaemic events after recent ACS in patients with persistent LDL-cholesterol elevation despite intensive or maximally tolerated statin therapy [51,52]. These trials support PCSK9 inhibition when conventional treatment is insufficient but do not establish routine administration to every patient during the index admission.

Evidence for very early PCSK9 inhibition is mainly lipid-related and mechanistic. PACMAN-AMI and HUYGENS showed favourable changes in non-culprit plaque burden and composition when alirocumab or evolocumab was added to intensive statin therapy [53,54]. However, these imaging studies were not powered to demonstrate reductions in cardiovascular death, recurrent myocardial infarction, or other major clinical outcomes. Their results support the biological rationale for rapid LDL-cholesterol lowering but should not be interpreted as proof of prognostic benefit from universal in-hospital PCSK9 inhibition.

Early PCSK9 inhibitor treatment may be considered in selected patients with markedly elevated LDL-cholesterol despite prior maximal therapy, probable familial hypercholesterolaemia, recurrent atherosclerotic events, documented statin intolerance, or values unlikely to reach target with statin–ezetimibe therapy alone [12,48]. Cost, access, treatment acceptance, and the likelihood of long-term adherence should also be considered.

European and North American recommendations share the principle of early intensive statin therapy but differ slightly in treatment thresholds. European guidance uses a target-based approach centred on LDL-cholesterol below 55 mg/dL, whereas the 2025 North American ACS guideline recommends adding non-statin therapy at LDL-cholesterol concentrations of at least 70 mg/dL and considers it reasonable between 55 and 69 mg/dL in patients receiving maximally tolerated statins [12,48].

Before discharge, the intended LDL-cholesterol goal, prescribed regimen, and timing of reassessment should be documented. Lipid concentrations and treatment tolerance should be reviewed within approximately 4–6 weeks, with prompt intensification when the goal has not been achieved [1,47]. Overall, high-intensity statin therapy and early ezetimibe when required are guideline-supported, whereas in-hospital PCSK9 inhibition remains a selective strategy for patients with particularly high residual lipid risk.

### 8.2. Residual Inflammatory Risk and Colchicine

Inflammation contributes to plaque instability and recurrent atherothrombotic events after ACS independently of LDL-cholesterol concentrations. The NLRP3 inflammasome–interleukin-1β–interleukin-6 pathway links innate immune activation with C-reactive protein production and atherosclerotic disease activity [55,56]. However, biological involvement does not imply that every anti-inflammatory intervention improves clinical outcomes.

CANTOS provided proof of principle that selective interleukin-1β inhibition can reduce recurrent cardiovascular events without affecting lipid levels, but the use of canakinumab was limited by cost, parenteral administration, and infection risk [57]. Greater attention has therefore focused on low-dose colchicine, an inexpensive oral treatment affecting neutrophil activity and inflammasome-related signalling.

The clinical evidence for colchicine is not uniform. COLCOT showed that treatment initiated within 30 days after myocardial infarction reduced its composite cardiovascular endpoint, mainly through fewer strokes and urgent revascularisations, without a significant reduction in cardiovascular death [58]. LoDoCo2 subsequently demonstrated benefit in stable chronic coronary disease, but its population and timing cannot be directly extrapolated to the acute post-infarction setting [59]. By contrast, CLEAR, the largest randomised trial of colchicine initiated after acute myocardial infarction, found no reduction in cardiovascular death, recurrent myocardial infarction, stroke, or unplanned ischaemia-driven revascularisation despite a reduction in C-reactive protein [60]. Diarrhoea occurred more frequently, while serious infections were not significantly increased.

Recent meta-analyses reflect this heterogeneity. Pooled analyses including chronic coronary disease and other vascular populations have reported reductions in major cardiovascular events, whereas analyses restricted to ACS have shown less consistent results and no clear reduction in mortality [61,62,63,64]. Differences in population, treatment timing, adherence, follow-up, and endpoint definitions may partly explain these findings. Colchicine should therefore not be described as either uniformly effective or ineffective across the full spectrum of coronary disease.

Treatment selection should also consider gastrointestinal intolerance, severe renal or hepatic dysfunction, and interactions with CYP3A4 or P-glycoprotein inhibitors. Myotoxicity may be relevant in patients receiving concomitant lipid-lowering therapy, particularly when renal function is impaired [65]. European and North American recommendations consequently support only selective consideration of low-dose colchicine rather than routine initiation after every ACS [1,66]. Although high-sensitivity C-reactive protein may identify persistent inflammatory activity, no prospectively validated biomarker-guided algorithm currently identifies patients who will benefit from treatment.

Overall, low-dose colchicine remains a selective strategy supported by inconsistent evidence and may be considered after evaluation of residual ischaemic risk, comorbidities, drug interactions, and treatment tolerability.

### 8.3. Cardiometabolic Therapies in Patients with Established Indications

Sodium–glucose cotransporter 2 (SGLT2) inhibitors and glucagon-like peptide-1 receptor agonists (GLP-1RAs) improve cardiovascular outcomes in selected patients with diabetes, heart failure, chronic kidney disease, or obesity. However, they should not be considered ACS-specific treatments, and their use after ACS should remain primarily based on established comorbid indications.

The early post-myocardial infarction role of SGLT2 inhibition has been assessed in DAPA-MI and EMPACT-MI. In DAPA-MI, dapagliflozin improved a hierarchical composite outcome, although the result was largely driven by cardiometabolic components rather than reductions in cardiovascular death or heart-failure hospitalisation [67]. In EMPACT-MI, empagliflozin initiated within 14 days after myocardial infarction did not significantly reduce the primary composite of first heart-failure hospitalisation or all-cause death; secondary analyses suggested fewer heart-failure admissions, while mortality was unchanged [68]. These trials do not support routine SGLT2 inhibitor initiation after every uncomplicated myocardial infarction but suggest a potential role in selected patients at increased risk of heart failure.

Evidence for GLP-1RAs in the immediate post-ACS setting is more limited. SELECT demonstrated that semaglutide reduced major cardiovascular events in patients with established cardiovascular disease and overweight or obesity without diabetes [69]. However, it was a long-term secondary-prevention trial and did not evaluate treatment initiated during an ACS admission. Its findings therefore support treatment of obesity as part of cardiovascular risk reduction but do not establish a specific role for GLP-1RAs in acute ACS care.

In clinical practice, SGLT2 inhibitors and GLP-1RAs may be initiated or continued after ACS when patients meet established cardiorenal or metabolic indications and are clinically stable. Their use should complement, rather than replace, treatments with proven ACS-specific benefit.

The current clinical status and applicability of the principal diagnostic and therapeutic strategies discussed in this review are summarised in Table 1.

## 9. Future Perspectives

Several diagnostic and therapeutic approaches may influence the future management of ACS, but their clinical maturity varies considerably. Future perspectives should therefore be discussed separately from interventions supported for routine practice. Promising biological mechanisms, favourable biomarker changes, or encouraging retrospective prediction models do not necessarily translate into improved patient outcomes. The following strategies remain investigational and require prospective confirmation before they can be incorporated into standard CICU pathways.

### 9.1. Targeted Anti-Inflammatory Therapies

Selective cytokine inhibition is a biologically plausible approach to residual inflammatory risk, but biomarker suppression cannot be assumed to translate into cardiovascular benefit. Interleukin-6 lies downstream of inflammasome and interleukin-1 signalling and contributes to hepatic C-reactive protein production, making it an attractive therapeutic target.

Ziltivekimab is a monoclonal antibody directed against the interleukin-6 ligand. In the phase 2 RESCUE trial, conducted in patients with chronic kidney disease and elevated high-sensitivity C-reactive protein, it produced marked dose-dependent reductions in inflammatory and thrombotic biomarkers without affecting LDL-cholesterol [70]. However, the study was not designed to assess cardiovascular outcomes.

Since the original submission, headline results from the phase 3 ZEUS trial have become available. In patients with established atherosclerotic cardiovascular disease, chronic kidney disease, and persistent inflammation, ziltivekimab did not reduce cardiovascular death, non-fatal myocardial infarction, or non-fatal stroke compared with placebo, despite the expected inhibition of the IL-6 pathway (hazard ratio 0.99; 95% confidence interval 0.88–1.11) [71]. Serious infections occurred more frequently, while all-cause mortality was similar. These results have not yet been published in full peer-reviewed form.

ZEUS did not specifically evaluate treatment initiated after acute myocardial infarction, but its findings weaken the assumption that substantial hsCRP reduction necessarily leads to fewer cardiovascular events. ARTEMIS is an ongoing phase 3 trial evaluating ziltivekimab after STEMI or NSTEMI in patients receiving contemporary standard treatment [72]. No outcome results are currently available.

Ziltivekimab should therefore remain classified as investigational. Other cytokine and immune pathways are at an earlier stage of development and currently have no established role in ACS management. Targeted anti-inflammatory therapies should remain confined to clinical trials until a favourable balance between cardiovascular efficacy and adverse effects has been demonstrated.

### 9.2. Artificial Intelligence, Advanced Imaging, and Multiomics

Artificial intelligence (AI) may support the interpretation of electrocardiographic, imaging, laboratory, haemodynamic, and electronic health record data in patients with suspected or confirmed ACS. Potential applications include recognition of acute coronary occlusion, risk stratification, prediction of clinical deterioration, and automated image analysis. However, diagnostic performance within a development dataset should not be equated with clinical usefulness or improved patient outcomes [73,74].

The ARISE pragmatic cluster-randomised trial provides an example of prospective implementation. AI-assisted electrocardiographic triage shortened treatment delays in patients with STEMI, including door-to-balloon and electrocardiogram-to-balloon times [75]. This demonstrates a potential workflow benefit but does not establish reductions in mortality, recurrent myocardial infarction, or heart failure, and requires confirmation in other healthcare systems. Other AI-enabled electrocardiographic models have shown encouraging diagnostic performance for coronary occlusion, but many remain retrospective and have not been tested as clinical decision tools [76].

Before routine implementation, AI models require external validation, calibration assessment, comparison with established clinical tools, and prospective evaluation within the intended workflow. Performance should be examined across sex, age, ethnicity, renal function, and different healthcare environments. Model drift, incomplete data, false alerts, limited interpretability, and uncertain responsibility for erroneous recommendations remain important barriers. AI should therefore augment rather than replace clinical judgement, particularly in the CICU, where treatment decisions depend on rapidly changing haemodynamics, competing diagnoses, procedural feasibility, bleeding risk, and patient preferences [77,78].

Advanced cardiovascular imaging already has established roles in selected ACS scenarios. The emerging component is the use of automated segmentation, quantitative plaque analysis, image fusion, and AI-assisted interpretation. These tools may improve reproducibility or efficiency, but evidence that they alter treatment selection or improve ACS outcomes remains limited [79].

Multiomics combines genomic, transcriptomic, proteomic, metabolomic, and other molecular data to identify biological phenotypes related to thrombosis, inflammation, myocardial injury, or ventricular remodelling. Current studies are mainly exploratory, involve heterogeneous or selected cohorts, and have not produced a prospectively validated strategy that improves treatment selection or clinical outcomes after ACS [80].

Future studies should therefore be multicentre and prospective, include representative populations, prespecify the clinical action triggered by the technology, and assess clinically meaningful outcomes rather than discrimination or surrogate measures alone. Until these requirements are met, AI-assisted decision support and automated imaging analysis should remain classified as evidence-evolving, whereas multiomics remains investigational.

The principal investigational or evidence-evolving approaches and their current limitations are summarised in Table 2.

## 10. Conclusions

Contemporary ACS management in the CICU requires the integration of rapid diagnosis, timely reperfusion or invasive assessment, appropriate antithrombotic therapy, and early recognition of haemodynamic, electrical, and mechanical complications. These priorities remain central across the full spectrum of ACS and should be adapted to the clinical presentation, coronary anatomy, bleeding risk, renal function, frailty, comorbidities, and the patient’s expected benefit from treatment.

Guideline-supported practice should remain clearly distinguished from strategies intended for selected clinical circumstances. Twelve-month DAPT remains the guideline-supported default after ACS in patients without high bleeding risk, whereas abbreviated regimens, de-escalation, and cangrelor are selective strategies. In patients requiring long-term oral anticoagulation, minimised triple therapy followed by dual therapy is guideline-supported, with treatment duration individualised according to bleeding and ischaemic risk.

High-intensity statin therapy, with early addition of ezetimibe when target attainment is unlikely, is guideline-supported, whereas early PCSK9 inhibition remains a selective strategy. Low-dose colchicine is a selective option supported by inconsistent evidence, while SGLT2 inhibitors and GLP-1 receptor agonists are established for specific heart failure, renal, metabolic, or obesity-related indications rather than as ACS-specific therapies.

Among evidence-evolving approaches, the clinical impact of hs-cTnT Gen 6 on patient flow and outcomes has not yet been established, and AI-assisted decision support requires further prospective validation. By contrast, therapies targeting microvascular obstruction and reperfusion injury, ziltivekimab, and multiomics remain investigational.

The contemporary CICU should therefore combine rigorous application of guideline-supported care with individualised use of selective strategies and critical evaluation of evidence-evolving and investigational strategies. Maintaining this distinction is essential to ensure that innovation complements, rather than precedes, treatments with demonstrated clinical benefit.

## Figures and Tables

**Table 1:** Current clinical status of selected strategies in acute coronary syndrome management.

Clinical Area	Strategy	Current Status	Application in ACS Care
Rapid diagnosis	Validated hs-cTn 0/1 h or 0/2 h algorithms [1,5,7]	Guideline-supported	Routine assessment of suspected NSTE-ACS, with results interpreted within the clinical context
Antiplatelet therapy	Aspirin plus an oral P2Y12 inhibitor for 12 months [1,12]	Guideline-supported	Standard after ACS in patients without high bleeding risk or another reason to modify treatment
Antiplatelet modification	Shortened DAPT, aspirin withdrawal, or de-escalation [16,17,18]	Selective	Appropriate in selected event-free patients according to bleeding and ischaemic risk
Intravenous platelet inhibition	Cangrelor [1,19]	Selective	P2Y12 inhibitor-naïve patients undergoing PCI when oral treatment cannot be administered or reliably absorbed
Anticoagulation	Acute parenteral anticoagulation and minimised triple therapy [1,12]	Guideline-supported	Agent and dose adapted to invasive strategy, renal function, bleeding risk, and the need for long-term oral anticoagulation
Revascularisation	Complete revascularisation in stable multivessel ACS [1,12,21,22,23]	Guideline-supported	Immediate or staged treatment individualised according to clinical stability, anatomy, renal function, and procedural complexity
Cardiogenic shock	Culprit-lesion PCI and selective temporary MCS [1,33,34,35,36]	Culprit-only initial PCI is guideline-supported; device use is selective	Routine multivessel PCI, IABP, or early VA-ECMO is not supported; microaxial support may be considered in selected patients
Lipid lowering	High-intensity statin with early ezetimibe when required [1,47,48,49,50]	Guideline-supported	Initiated or intensified during hospitalisation to achieve rapid LDL-cholesterol reduction
PCSK9 inhibition	Early monoclonal antibody therapy [48,51,52,53,54]	Selective	Considered in patients with markedly elevated LDL-cholesterol, prior maximal therapy, familial hypercholesterolaemia, or very high residual lipid risk
Residual inflammation	Low-dose colchicine [1,58,60,61,62,63,64,66]	Selective	Possible option after individual assessment; routine administration after acute myocardial infarction is not supported; evidence is inconsistent
Cardiometabolic treatment	SGLT2 inhibitors and GLP-1RAs [67,68,69]	Guideline-supported for specific comorbid indications	Used according to diabetes, heart failure, CKD, or obesity rather than as routine ACS-specific therapy

**Note:** “Guideline-supported” indicates an intervention recommended for a broad eligible ACS population. “Selective” indicates a strategy requiring individual assessment of clinical presentation, ischaemic and bleeding risks, comorbidities, anatomy, or treatment feasibility. “Evidence evolving” indicates that diagnostic, mechanistic, or preliminary clinical evidence is available but routine implementation has not been established. Abbreviations: ACS, acute coronary syndrome; CKD, chronic kidney disease; DAPT, dual antiplatelet therapy; GLP-1RA, glucagon-like peptide-1 receptor agonist; hs-cTn, high-sensitivity cardiac troponin; IABP, intra-aortic balloon pump; MCS, mechanical circulatory support; NSTE-ACS, non-ST-segment elevation acute coronary syndrome; PCI, percutaneous coronary intervention; PCSK9, proprotein convertase subtilisin/kexin type 9; SGLT2, sodium–glucose cotransporter 2; VA-ECMO, venoarterial extracorporeal membrane oxygenation.

**Table 2:** Investigational and evidence-evolving approaches in acute coronary syndrome management.

Emerging Approach	Current Evidence	Present Clinical Status	Evidence Still Required
hs-cTnT Gen 6	Analytical and diagnostic validation studies [8,9]	Evidence evolving	Prospective implementation studies evaluating sampling, length of stay, clinical decisions, safety, and outcomes
Intracoronary fibrinolysis for MVO	Randomised trials have not demonstrated consistent improvement in myocardial reperfusion or clinical events [25,26]	Investigational	Identification of responsive phenotypes and demonstration of benefit on heart failure, recurrent events, or mortality
Other treatments targeting reperfusion injury	Mainly mechanistic, imaging-based, or neutral randomised studies [27,28,29,30,31]	Investigational	Reproducible reduction in infarct size or MVO followed by improvement in clinical outcomes
Ziltivekimab	Marked biomarker reduction in phase 2 [70]; neutral headline outcome results in ZEUS [71]; ARTEMIS ongoing after myocardial infarction [72]	Investigational	Complete safety data and randomised evidence of cardiovascular benefit in the post-ACS population
AI-assisted ECG triage	Reduced treatment times in the pragmatic ARISE trial [75]; high diagnostic performance for coronary occlusion in retrospective international validation [76]	Evidence evolving	External validation, prospective implementation, assessment of bias, and demonstration of clinical benefit
AI-assisted imaging	Automated segmentation and quantitative image interpretation [79]	Evidence evolving	Demonstration that automated analysis changes treatment selection or improves outcomes
Multiomics-based phenotyping	Exploratory and heterogeneous cohort studies [80]	Investigational	Standardised platforms, reproducible patient subgroups, actionable treatment selection, and prospective outcome validation

**Note:** Inclusion in this table does not imply current suitability for routine clinical use. Improvement in surrogate endpoints, biomarker reduction, or diagnostic discrimination should not be interpreted as evidence of improved cardiovascular outcomes. Abbreviations: ACS, acute coronary syndrome; AI, artificial intelligence; ECG, electrocardiogram; hs-cTnT, high-sensitivity cardiac troponin T; MVO, microvascular obstruction.

## Data Availability

No new data were created or analysed in this study. Data sharing is not applicable to this article.
